# Acute Effects of Facial Coverings on Anaerobic Exercise Performance in College-Aged Adults

**DOI:** 10.3390/ijerph191710500

**Published:** 2022-08-23

**Authors:** Ryan T. Conners, Paul N. Whitehead, Thomas Skarp, Briana Waller, Mark Richard, Carrington Bain, Megan Monks, Mark A. Faghy

**Affiliations:** 1 Department of Kinesiology, The University of Alabama in Huntsville, Huntsville, AL 35899, USA; 2 School of Human Sciences, University of Derby, Derby DE22 1GB, UK

**Keywords:** facial coverings, anaerobic exercise, SHEMA97, surgical mask, 300-yard shuttle, exercise performance

## Abstract

The use of facial coverings has been amplified during the COVID-19 pandemic as a means to minimize the spread of disease. However, facial coverings may impede ventilation during high-intensity activity, leading to a reduction in cardiopulmonary exercise capacity. Thus, the purpose of this study was to determine the acute impact of different facial coverings on exercise performance in college-aged individuals during a 300-yard shuttle. It was hypothesized that the lowest heart rate (HR), completion time (CT), and rate of perceived exertion (RPE) would occur with no mask. Furthermore, it was hypothesized the SHEMA97 mask would have lower HR, CT, and RPE compared to surgical and fabric masks. Results showed the use of the fabric mask resulted in significantly higher HR compared to no mask (*p* = 0.006). The SHEMA97 mask resulted in faster CT and lower RPE compared to both the fabric and surgical masks (*p* < 0.001). All mask conditions yielded significantly higher levels of perceived discomfort than wearing no mask (*p* < 0.05). While the use of facial coverings can help prevent the spread of disease, their use during exercise may pose limitations to performance; however, the ability of the SHEMA97 to provide minimal changes to CT and RPE provides a promising option.

## 1. Introduction

The coronavirus disease 2019 (COVID-19) pandemic has been caused by widespread transmission and infection of the SARS-CoV-2 virus [1]. Emerging variants of concern and altered viral properties have led to an increased risk of infection, which is transmitted from person to person through virus-carrying respiratory droplets [2]. Transmission can take place during speaking, sneezing, coughing, or forced exhalation during exercise [2]. An infection with COVID-19 is associated with individuals experiencing acute symptoms such as fever, fatigue, and nausea [3,4,5,6]. However, severe cases of COVID-19 have caused pulmonary edema, multiple organ dysfunction, and widespread disability and mortality [3,4,7,8,9,10]. Thus, COVID-19 has taken a large toll on global health and with continued risk of future variants and spikes in transmission, countries/governing bodies are developing strategies to reduce the transmission [11]. Due to COVID-19 primarily being spread through the transmission of respiratory particles [7], one of the most common strategies utilized by governments/legislative bodies is the recommendation or mandatory wearing of facial coverings [12,13].

Facial coverings reduce COVID-19 transmission and infection by preventing infected particles from being exhaled into the environment by the host or inhaled by a recipient and have been responsible for reducing viral spread and saving lives [14]. However, there are various types of facial coverings, including those made from different types of fabric (i.e., surgical masks, fabric masks, and available commercial products) [15]. Despite the different fabrics used to create the masks, they are constructed to protect against respiratory droplets and particles [6]. Two of the most common facial coverings utilized are surgical masks and fabric masks [16]. Fabric masks can be homemade and are not “leakproof” [16]. Fabric masks are also created for everyday use and provide a decreased risk for droplet transmission during exhalation. They are commonly recommended for the general population and are usually less expensive compared to alternative facial coverings (i.e., surgical masks or filtering facepieces) [15]. On the other hand, surgical masks abide to strict guidelines to provide a higher level of protection against infection [15]. Surgical masks provide filtering capability, can be utilized for daily use, and provide patient protection. In addition, there has been a commercial drive by manufacturers leading to widespread availability of face coverings that use a variety of materials that have been created with limited knowledge on their efficacy.

One of the more popular facial coverings being utilized in collegiate and professional athletics is the SHEMA97 reusable functional active mask (HelmetFitting.com). The SHEMA97 is a Food and Drug Administration approved mask that is breathable, ultralight, and is antiviral [17]. The SHEMA97 and other facial coverings are produced due to athletes and coaches having to wear facial coverings during exercise, practice, and/or competitions. While some types of facial coverings provide higher amounts of reduction in the spread of transmission, there have also been reports that they may also hinder physical activity and/or exercise by increased physiological and perceptual responses [18]. Previous research suggests that wearing facial coverings while engaging in exercise could potentially affect the individual’s breathing ability by increasing the inspiratory and expiratory pressures required to achieve ventilation [19], which has been shown to reduce cardiopulmonary exercise capacity [20].

The results of the study by Fikenzer and colleagues [20] indicated that wearing a nose and mouth facial covering impacted ventilation (−23%) and maximum aerobic capacity (−13%) while performing high-intensity aerobic exercise compared to a no mask condition. Furthermore, research by Kampert et al. [21] indicated that surgical masks worn while at rest resulted in decreased lung function compared to a no mask condition. As a result, there could be important considerations for athletes and coaches to mitigate reductions in performance whilst adhering to imposed safety guidelines and precautions while wearing facial coverings [21]. During exercise participation, the physiological impact of facial coverings compared to the protective effect needs to be considered in detail while performing exercise [20,21].

In addition to the physiological impact of facial coverings on aerobic exercise, there has been minimal research on the impact of different facial coverings on anaerobic exercise performance. The 300-yard shuttle run is a widely used reliable test and is recognized by the National Strength and Conditioning Association for assessing anaerobic exercise capacity [22,23,24]. The test includes two 300-yard anaerobic exercise bouts separated by a 5 min rest period [22]. The 300-yard shuttle test incorporates short-duration high-intensity movements and mimics energy expenditure bouts seen during anaerobic-based sport participation [24]. In addition to aerobic exercise sports, anerobic exercise dominant sports are also being impacted by guidelines and rules requiring the wearing of facial coverings. Thus, the purpose of this study was to determine the acute impact of different facial coverings on anaerobic exercise performance in college-aged individuals. It was hypothesized that the no mask condition would result in the lowest heart rate (HR), completion time (CT), and rate of perceived exertion (RPE) values. Furthermore, it was hypothesized that the SHEMA97 mask would have lower HR, CT, and RPE values compared to the surgical and fabric masks. Lastly, it was hypothesized that the fabric mask would have the highest HR, slowest CT, and highest RPE values compared to the other facial coverings.

## 2. Materials and Methods

### 2.1. Subjects

A total of 26 (16 males and 10 females) participants were recruited for participation in the study. Participants included active college-aged adults from a University in the Southeastern United States, which included participation in aerobic exercise ≥60 min a day for ≥3 days per week [25,26]. Participants were not required to have previous exercise experience while wearing a facial covering to participate in the study. Baseline participant characteristics are listed in Table 1. The sample size was utilized for the study to be sufficiently powered (α = 0.05, β = 0.80, medium effect 0.5; G*Power, version 3.1.9.3, Dusseldorf, Germany). Prior to the commencement of the study, participants were excluded from the study if they had a history of cardiovascular disease, pulmonary disorders, metabolic diseases, or were smokers [27]. All research procedures were approved by The University of Alabama in Huntsville Institutional Review Board (EE2020113) and conformed to the standards set by the Declaration of Helsinki. Written informed consent was completed by each participant prior to participation in the study. Each participant was instructed not to participate in vigorous physical activity 12 h prior to each exercise session [27]. Furthermore, each participant was instructed not to consume food or caffeine 2 h prior to each exercise testing session. A minimum of 24 h of mandatory rest was completed in-between each exercise session. Participants completed each testing session during the same time of the day (± 2 h) and during similar temperature conditions (±2 °F).

### 2.2. Facial Coverings

**Figure 1:**
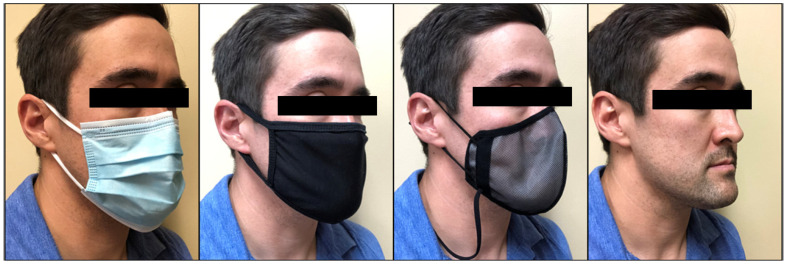
Facial coverings worn during the anaerobic exercise bouts (surgical, fabric, SHEMA97, and no mask condition).

Our study utilized four separate facial covering conditions: surgical mask, fabric facemask, SHEMA97, and a no mask condition (see Figure 1). The three masks are detailed below. Surgical mask (USA ASTM F2100): The surgical mask was 3.7 × 6.9 in and weighed a total of 3.2 g. The dead space of the surgical mass was dependent on the wearing fitting. The surgical mask consisted of 3 layers and the materials are industry-standard for a surgical mask. The surgical mask provided level 1 protection and a filtration of >95% for 3 μm and 0.1 μm particles. Fabric facemask: Champion (Rural Hall, North Carolina). The fabric facemask was 50% cotton and 50% polyester. The mask consisted of a total of 2 layers and was 5.5 × 8.5 in. The total mass of the fabric facemask was 17.3 g. The overall dead space for the facemask depended on the wearer and fitting. SHEMA97: Helmetfitting.com (Rushan Longma Garments Co, Seoul, South Korea) The SHEMA97 is an FDA-registered (Listing #D417280) product and comprises a nano filter, protective membrane mesh, and polymeric resin. The total mass of the SHEMA97 was 6 g. The sizes of the mask range from a medium = 8 × 5 in to a 2 × l = 10 × 6.5 in. The overall dead space for the facemask depended on the wearer and fitting. The SHEMA97 is antibacterial and had a filtration of >97% for 3 μm and 0.1 μm particles.

### 2.3. Methods

Before arrival, participants were contacted and confirmed that they had not been exposed to anyone with a confirmed case of COVID-19, along with not currently testing positive for COVID-19. When the participant arrived at the indoor basketball court, they had their temperature taken as a safety precaution to help avoid the spread of COVID-19. Once a participant completed the informed consent process, they were fitted with a polar heart rate monitor (T31, Kempele, Finland) and watch. The heart rate monitor was placed directly on the skin at the level of the xiphoid process [28,29]. After the heart rate monitor was attached, a baseline heart rate measure was taken for each participant, following five minutes of seated rest. Following the baseline heart rate measure, participants completed a dynamic warmup. The dynamic warmup consisted of jumping jacks, butt kickers, walking leg hugs, straight leg walking high kicks, karaoke, walking lunges with a twist, and body weight squats [30].

The order of the facial coverings worn by each participant was randomized for each trial. A random number generator was used to determine the sequence of facial coverings that was utilized for each participant. Following the standardized warm-up, the facial covering condition for that day was told to the participant. The no mask condition was completed without any facial covering. If a facial covering condition was selected, the facemask was put on by the participant and was checked by a trained member of the research team, to make sure it was secured correctly according to manufacturers’ specifications [27]. Each facial covering was worn for the entire duration of the 300-yard shuttle test (first anaerobic exercise test), the five-minute rest period, and the second 300-yard shuttle bout. The 300-yard shuttle test was utilized based upon the test incorporating two anaerobic exercise bouts that are separated by a five-minute aerobic recovery.

The 300-yard shuttle test protocol required measuring and marking both a start and stop reference point that were 25 yards apart on an indoor basketball court [22]. The participant began the test behind the starting line. At the start of the stopwatch, the participant ran to the stop line and was required to touch it with his or her foot before returning to the starting line. This was completed a total of 6 times (50 yards total × 6 reps = 300 yards) without stopping. The time taken to complete the 300-yard shuttle (CT) was recorded via stopwatch. Immediately after completing the 300-yard shuttle, the participants’ HR was read from the watch and was recorded. A separate stopwatch was then started for the five-minute period of rest before a second 300-yard shuttle bout was completed. To eliminate delayed starts between the resting period and the start of the second round, participants were at the starting line when thirty seconds remained in the rest period. The overall CT score was calculated by averaging the CT for the two 300-yard shuttles that were completed for each facial covering session.

Each participant completed the 300-yard shuttle run on four different occasions with a 24-to-48-h rest between each trial. During each trial, participants were instructed and verbally encouraged to complete each trial as fast as possible and to give consistent effort during the 300-yard shuttles across the four conditions [22,23]. Each facial covering was worn for the duration of the 300-yard shuttle test, the five-minute rest period, and the second 300-yaard shuttle bout. Following each facial covering exercise trial, the participants’ RPE was measured. The RPE value was measured on a 1–10 scale, where 1 was the equivalent of “extremely easy exercise” and a value of 10 was “so hard cannot continue” [31]. After completing RPE, the participant filled out a subjective discomfort survey that was created based upon the questions utilized in the study by Li et al. [32]. The survey consisted of 5 questions, which asked about the amount of humidity, heat, breathing resistance, fatigue, and overall discomfort while wearing the specific facial covering for each trial [32]. Each question was scored on a 1–10 Likert scale and the individual scores were recorded for each facial covering testing session.

### 2.4. Statistical Analysis

For all statistical analyses, the Statistical Package for Social Sciences (SPSS Version 26, Armonk, NY, USA) was used. Normality of all HR, CT, RPE, and discomfort scale measurements was assessed using the Shapiro–Wilk test [33]. The impact of each facial covering on HR, CT, RPE, and discomfort survey values was assessed using repeated measures analysis of variances (RMANOVA). Effect sizes were calculated as partial eta squared (*η_p_*^2^), which provided information on the magnitude of the difference between the facial coverings. The threshold values for *η_p_*^2^ were defined as small (0.01), moderate (0.06), and large effects (0.14) [34]. Pairwise comparisons were utilized to further analyze significant differences in HR, CT, and discomfort values across the mask conditions. The level of significance was set at *p* < 0.05 for all analyses.

## 3. Results

All HR, CT, and RPE data based upon mask condition are listed in Table 2. Heart rate (*F* = 3.67, *p* = 0.016, *η_p_*^2^ = 0.128), CT (*F* = 23.73, *p* < 0.001, *η_p_*^2^ = 0.487), and RPE (*F* = 142.42, *p* < 0.001, *η_p_*^2^ = 0.851) were all significantly different across the facial covering conditions. Pairwise comparisons revealed the fabric mask resulted in significantly higher HR compared to the no mask condition (*p* = 0.006). Completion time values were faster when wearing the SHEMA97 compared to the fabric (*p* < 0.001) and surgical mask (*p* < 0.001) conditions. Additionally, the no mask condition resulted in the fastest CT compared to the SHEMA97, fabric, and surgical mask conditions (*p* < 0.05). There was no difference in CT between the fabric and surgical mask (*p* = 1.00). Rate of perceived exertion was the lowest in the no mask condition compared to the other facial covering conditions (*p* > 0.05). The SHEMA97 condition resulted in lower RPE values compared to both the fabric (*p* < 0.001) and surgical mask (*p* < 0.001) conditions. There were no significant differences in RPE between the surgical mask and fabric mask conditions (*p* = 1.00).

All discomfort survey scores based upon the facial coverings worn are listed in Table 3. The results of the study indicated significant differences in humidity (*F* = 53.69, *p* < 0.001, *η_p_*^2^ = 0.682), heat (*F* = 53.45, *p* < 0.001, *η_p_*^2^ = 0.680), breathing resistance (*F* = 87.33, *p* < 0.001, *η_p_*^2^ = 0.770), fatigue (*F* = 174.31, *p* = < 0.001, *η_p_^2^* = 0.409), and overall discomfort (*F* = 52.29, *p* < 0.001, *η_p_*^2^ = 0.677) based upon the facial covering worn. The no mask condition resulted in significantly lower humidity, heat, breathing resistance, fatigue, and overall discomfort compared to the SHEMA97, fabric, and surgical mask conditions (*p* < 0.05). The SHEMA97 mask condition resulted in significantly lower humidity, heat, breathing resistance, fatigue, and overall discomfort values compared to both the fabric and surgical mask conditions (*p* < 0.05). There were no significant differences between the fabric and surgical masks for all five of the discomfort survey questions (*p* > 0.05).

## 4. Discussion

The purpose of this study was to determine differences in acute anaerobic exercise performance by comparing HR, CT, RPE, and discomfort survey results between various facial coverings. Our findings indicated that the facial covering worn (SHEMA97, fabric, surgical) had a significant impact on HR, CT, RPE, and discomfort survey results in healthy college-aged individuals. As a result, our hypothesis that the no mask condition would have limited impact on anaerobic exercise performance, physiological strain and perceptual responses was supported. Furthermore, the hypothesis that the SHEMA97 facial covering would have lower HR, CT, and RPE values when compared to fabric and surgical mask conditions was supported. The hypothesis that the fabric mask would have the highest HR, CT, and RPE values was not supported, as the results of the study indicated that surgical and fabric masks had similar HR, CT, and RPE values.

Heart rate values for each facial covering condition are listed in Table 2. Relative and absolute change in HR values was similar when performing the 300-yard shuttle test while wearing the different types of facial coverings. The results are similar to the findings of a study performed by Woolf, Bidwell, and Carlson [35], who also showed increases in HR when college-aged adults performed anaerobic exercise. However, HR was significantly higher in the fabric mask condition compared to the no mask condition (*p* = 0.006). These findings are in agreement with the research conducted by Roberge, Kim, and Benson [36], who identified that surgical masks are associated with clinically significant increases in HR, compared to rest, when completing moderate exercise activities. The current study’s findings are in contrast to the results of a study by Shaw et al. [37], who identified no differences in HR between a no mask, surgical mask, and fabric mask while performing vigorous exercise in health adults. A potential explanation of why there was no change in HR between the no mask condition and the SHEMA97 and surgical masks is the duration of exercises was not sufficient to cause significant changes in HR [36]. The difference in HR between fabric masks and no mask conditions could be due to the thickness of the material of the fabric mask compared to the other facial coverings. Thicker facial coverings may cause an increase in relative physiologic workload, increasing anaerobic metabolic activity resulting in an acidic environment at the alveoli and at the blood vessels, which would result in physiological alterations, such as cardiac overload [38]. Fabric masks may also impair thermoregulation, discomfort, and increased hypoxia compared to the no mask condition [39,40]. Thus, no mask, SHEMA97, or surgical mask may be the optimal choices for preventing the spread of the virus and limiting impact on HR while performing anaerobic exercise [41,42].

Like HR, CT was also impacted by the type of facial covering that was worn by the college-aged participants. Both the no mask and SHEMA97 conditions resulted in lower CT compared to the surgical and fabric mask conditions. The similar CT values between the no mask and SHEMA97 facial covering was also found in research by Shaw et al. [37], which found no impact on exercise performance with the wearing of a surgical mask or a N95 respirator mask. The CT results are also in line with the findings of the meta-analysis by Shaw et al. [42], who indicated there was minimal impact on exercise performance while wearing a face mask. The lack of effect on CT with the SHEMA97 mask potentially indicates that the design and materials of the mask do not reduce breathing capacity during high-intensity anaerobic exercise. As a result, the use of the SHEMA97 face mask may provide adequate protection while exercising indoors, whilst not impeding exercise performance where there are issues of smaller spacing, close proximity to other athletes, and increased breathing during exercise [42,43,44,45].

Completion time (anaerobic exercise performance) was negatively impacted in both the surgical and fabric facial covering conditions. This is in agreement with the research performed by Tornero-Aguilera, Rubio-Zarapuz, Bustamante-Sanchez, and Clemente Suarez [39], which also showed decreases in anaerobic running performance while wearing a surgical mask. The decrease in performance is suggested to be due to the metabolic shift and a decrease in efficiency, which occurs due to the impaired autonomic stability and lowered blood supply during the exercise bout [38,39]. It could also be due to increased RPE, dyspnea, and lower blood oxygenation, which are commonly reported while exercising with a facial covering [39,46]. However, the negative impact of both the surgical and fabric masks are in contrast to the findings of the metanalysis by Shaw et al. [42].

In addition to CT, RPE was also higher when wearing a facial covering compared to the no mask condition. These findings are similar to the study by Tornero-Aguilera et al. [39], which also found increases in RPE while wearing a mask and performing anaerobic exercise (50 m and 400 m sprint tests). The increased RPE is more than likely due to the increased feelings of heat, breathing resistance, and overall discomfort that were identified in the discomfort survey. Research studies by Tornero-Aguilera et al. [39] and Fikenzer et al. [20] also showed increases in RPE due to the discomfort and high amount of perception and stress seen during intense exercise. The changes in RPE during exercise while wearing facial coverings, compared to a no mask condition, are in contrast to the findings of multiple research studies [37,47,48,49]. These studies found no significant impact of wearing a facial covering on participant RPE. However, the majority of these studies involved light to moderate levels of exercise and cycling as the mode of exercise [37,47,48,49]. Thus, the exercise mode and level of exercise intensity utilized in these studies may have resulted in no significant changes in RPE. Since previous research with facial coverings and exercise has found significant differences in RPE, the study incorporated a modified discomfort survey that was completed after each trial. Results of the study indicated that there were significant differences in humidity, heat, breathing resistance, fatigue, and overall discomfort while wearing the facial coverings compared to not wearing a mask at all. This echoes the findings of researchers [20,39,50,51,52] who have investigated the impact of facial coverings on both aerobic and anaerobic exercise performance. The overall levels of discomfort while wearing the facial coverings coincides with the increased RPE values. Thus, the wearing of the masks could have resulted in increased stress, impaired thermoregulation, discomfort, and increased levels of hypoxia [20,39,50].

Although perceptual discomfort was increased when wearing a facial covering compared to a no mask condition, the SHEMA97 had lower survey values compared to the surgical and fabric mask conditions. This is similar to the results of research performed by Fikenzer et al. [20], which showed significant lower values in heat, breathing resistance, and overall discomfort with a surgical mask compared to a N95 respirator mask. Although the SHEMA97 (6.0 g) is slightly heavier than the surgical mask (3.2 g), participants indicated that it was the most comfortable out of the different types of facial coverings. As a result, the participants may have felt that the SHEMA97 was less subjectively disturbing to wear and would not have had such an impact on exercise performance [20]. The materials used for the different facial coverings may not have only impacted the participants’ level of discomfort but may have also impaired their physical exercise performance.

One of the limits of the study was that the population was healthy college-aged individuals. As a result, the individuals may have different exercise training regimens, previous levels of exercise experience, and amount of time spent performing high-intensity anaerobic exercise. Additionally, the study did not utilize blood oxygen saturation testing during the research study. The research study also did not control for hormone (cortisol and/or alpha amylase) or hydration level amongst the research participants. However, the researchers do propose the use of hormonal testing for future research. Additionally, the inclusion of KN95 or respirator masks would be a recommendation for future research. Future research could also incorporate high-level athletes and determine if the level of training impacts anaerobic performance while wearing facial coverings in both aerobic and anaerobic exercise.

## 5. Conclusions

Facial coverings are being worn to decrease the spread of COVID-19. However, the wearing of facial coverings results in increased HR, CT, and RPE when compared to not wearing a mask while completing anaerobic exercise. Wearing facial coverings while performing anaerobic exercise also results in increased feelings of heat, breathing resistance, and overall discomfort. This can potentially impact athletes and non-athletes while they are performing high-intensity anaerobic exercise while wearing a facial covering. This may impact legislation and association rules of mandating the wearing of facial coverings while exercise or exercising indoors.

## Figures and Tables

**Table 1:** Participant baseline characteristics.

Variable	Total (*n* = 26)	Male (*n* = 16)	Female (*n* = 10)
Age (years)	21.2 ± 1.6	21.6 ± 2.4	20.9 ± 1.0
Height (cm)	169.4 ± 10.8	178.6 ± 10.9	164.6 ± 7.2
Body mass (kg)	73.5 ± 20.35	77.5 ± 14.5	71.4 ± 23.0
Baseline heart rate (bpm)	71.0 ± 10.1	70.8 ± 11.5	71.1 ± 9.7

Note. Values are mean ± standard deviation.

**Table 2:** Performance variables based upon mask condition.

Variable	Mask Condition				Pairwise Comparisons (*p* < 0.05)
	SHEMA97	Fabric	Surgical	No Mask	
C.T. (s)	75.15 ± 13.30	77.94 ± 13.37	77.82 ± 13.87	73.56 ± 13.95	*a*, *b*, *c*, *e*, *f*
Heart rate (bpm)	126.50 ± 19.28	131.38 ± 23.11	130.03 ± 22.08	125.65 ± 19.88	*e*
RPE	5.77 ± 1.42	7.54 ± 1.24	7.77 ± 1.37	2.92 ± 1.29	*a*, *b*, *c*, *e*, *f*

Note. RPE = Rate of perceived exertion; C.T. = completion time; *a* = SHEMA97 vs. Fabric; *b* = SHEMA97 vs. Surgical; *c* = SHEMA97 vs. No Mask, *d* = Fabric vs. Surgical; *e* = Fabric vs. No Mask; *f* = Surgical vs. No Mask.

**Table 3:** Measures of discomfort.

Variable	Mask Condition				Pairwise Comparisons (*p* < 0.05)
	SHEMA97	Fabric	Surgical	No Mask	
Humid	3.62 ± 1.55	5.19 ± 1.67	5.65 ± 1.72	1.58 ± 1.03	*a*, *b*, *c*, *d*, *e*
Hot	4.00 ± 1.50	5.88 ± 1.45	6.2 ± 1.68	2.27 ± 1.40	*a*, *b*, *c*, *d*, *e*
Breathing Resistance	3.92 ± 1.74	6.50 ± 1.75	7.19 ± 1.47	1.85 ± 1.05	*a*, *b*, *c*, *d*, *e*
Fatigue	5.04 ± 1.59	7.00 ± 1.20	6.54 ± 1.63	4.73 ± 1.56	*a*, *b*, *d*, *e*
Overall Discomfort	4.46 ± 1.36	6.42 ± 1.75	7.27 ± 1.43	2.92 ± 1.57	*a*, *b*, *c*, *d*, *e*

Note. *a* = SHEMA97 vs. Fabric; *b* = SHEMA97 vs. Surgical; *c* = SHEMA97 vs. No Mask, *d* = Fabric vs. No Mask; *e* = Surgical vs. No Mask.

## Data Availability

Not applicable.
